# Nepetin reduces virulence factors expression by targeting ClpP against MRSA-induced pneumonia infection

**DOI:** 10.1080/21505594.2022.2051313

**Published:** 2022-04-01

**Authors:** Shisong Jing, Xinran Ren, Li Wang, Xiangri Kong, Xingye Wang, Xiren Chang, Xuerui Guo, Yan Shi, Jiyu Guan, Tiedong Wang, Bingmei Wang, Wu Song, Yicheng Zhao

**Affiliations:** a Clinical Medical College, Changchun University of Chinese Medicine, Changchun, China; b School of Pharmaceutical Science, Jilin University, Changchun, China; c Affiliated Hospital to Changchun University of Chinese Medicine, Changchun University of Chinese Medicine, Changchun, China; d College of integrated Chinese and Western medicine, College of rehabilitation, Changchun University of Chinese Medicine, Changchun, China; e Key Laboratory of Zoonosis, Ministry of Education, College of Veterinary Medicine, Jilin University, Changchun, China; f College of Animal Science, Jilin University, Changchun, China

**Keywords:** MRSA, anti-virulence, inhibitor, caseinolytic peptidase P, pneumonia

## Abstract

The resistance of *Staphylococcus aureus* (*S. aureus*) to various antibiotics has increased dramatically due to the misuse of antibiotics, and thus the development of new anti-infective drugs with new targets is urgently needed to combat resistance. Caseinolytic peptidase P is a case in hydrolase that regulates the virulence level of *S. aureus*. Here, we found that nepetin, a small-molecule compound from traditional Chinese herbal flavonoids, effectively inhibits ClpP activity. Nepetin suppressed the virulence of *S. aureus* and effectively combated the lethal pneumonia caused by MRSA. The results of cellular thermal shift assay showed that nepetin could bind to ClpP and reduce the thermal stability of ClpP, and the *K*_*D*_ value of 602 nM between them was determined using localized surface plasmon resonance. The binding mode of nepetin and ClpP was further investigated by molecular docking, and it was found that Ser-22 and Gln-47 of ClpP residues were found to be involved in the binding of nepetin to ClpP. In conclusion, we determined that nepetin is a ClpP inhibitor and an effective lead compound for the development of a virulence factor-based treatment for MRSA infection.

## Introduction

*Staphylococcus aureus* causes a spectrum of diseases, including superficial skin infections to serious pneumonia [1]. The misuse of antibiotics has led to the emergence of methicillin-resistant *Staphylococcus aureus* (MRSA), making *S. aureus* infections increasingly difficult to treat and resulting in high morbidity and mortality rates [2]. On the other hand, antibiotic resistance may spread and proliferate among different opportunistic pathogenic bacteria, subsequently promote the evolution of antibiotic resistance [3]. Therefore, the development of new strategies and targets for bacterial resistance is urgently needed.

S.*aureus* infection relies on the various virulence factors it produces, for example, *hla*, a toxin secreted by most pathogenic *S. aureus*, binds to target cell membranes and causes cellular damage [4]. Panton-Valentine leukocidin (PVL) kills many immune cells, such as neutrophils, macrophages and lymphocytes, and induces an inflammatory response that promotes the invasion of pathogenic bacteria [5]. Antivirulence drugs that target virulence factors to reduce the virulence of pathogens, rather than eliminating them, thus avoiding the selective pressure imposed by drugs. Another advantage of the anti-virulence strategy compared to conventional antibiotics is that it avoids the killing of normal microorganisms and promotes the immune process of the host [6].

The expression of virulence in bacteria is a highly correlated and coordinated process that is mediated by known or unknown regulatory factors. Thus, inhibiting certain virulence factors may lead to upregulation of other virulence factors [7,8]. Various studies have established that although *agr* mutants eliminate the expression of α-toxin, they also cause a surge in the expression of *staphylococcal* protein A (SpA) [9]. Therefore, *S. aureus* infection is rarely achieved by suppressing a single virulence factor, and simultaneous suppression of the expression of multiple virulence genes or virulence regulators would be a very promising strategy. The core role of *S. aureus* virulence regulation is attributed to the caseinolytic peptidase P (ClpP) protein, a highly conserved serine protease essential for the virulence of a variety of pathogens [10]. The virulence of the mutant *clpP S. aureus* was significantly reduced, which may be achieved by upregulating several transcriptional repressors of the *sarA* family and downregulating the *agr* quorum-sensing system [11,12]. The pathogenicity of the bacteria in which *clpP* was knocked out in infected mice was significantly weakened [10]. Due to the important regulatory effect of ClpP on the virulence of pathogenic bacteria, it has become a promising new antibacterial candidate target.

A large number of bioactive compounds extracted from traditional herbal medicines have significant antibacterial activity and have been widely used as alternative medicines for the treatment of infectious diseases [13]. Herbal medicines naturally produce a variety of secondary metabolites that can defend against infestation brought about by microorganisms in the environment [14]. Currently, many herbal monomeric small molecule compounds have been shown to be effective in inhibiting bacterial virulence, for example, licochalcone A can reduce toxin secretion by *S. aureus* by inhibiting agrA [15]. Luteolin can effectively reduce the expression of α-toxin and decrease the pathogenicity of *S. aureus* [16]. Therefore, traditional herbal monomer compounds are a viable source of antibacterial hit compounds. ClpP is of great research interest as a newly emerged anti-virulence target. Compounds of *β*-lactones and phenyl esters could inhibit the activity of ClpP and thus effectively reduce the pathogenicity of *S. aureus* [17,18]. In addition, Gao et al. found that a small molecule compound, M21, could reduce most of the virulence factors in *S. aureus* by inhibiting ClpP and was effective against lethal systemic infections caused by MRSA in a mouse infection model [8]. However, the number of ClpP inhibitors found so far is still small and not yet applied in the clinic, so the search for inhibitors of ClpP is still of great importance.

In the present study, by screening our chemical library of small molecules from natural products, we found that nepetin reduced the expression levels of multiple virulence factors in MRSA by inhibiting the activity of ClpP. Nepetin is a flavonoid extracted from the plant *Eupatorium adenophorum* that exerts a significant anti-inflammatory effect, especially on the development of osteoarthritis [19,20]. We showed that nepetin binds ClpP and reduces its thermal stability and determined the affinity constant between the two at 602 nM. Meanwhile, we simulated the binding mode and verified the key binding sites between nepetin and ClpP. Most importantly, nepetin is effective at protecting mice from lethal pneumonia caused by MRSA. Based on these results, nepetin may be a potential agent for use against MRSA infections.

## Methods

### Bacterial strains, plasmids and reagents

The MRSA strain USA300 and MSSA strain Newman have been described previously [21]. Unless indicated otherwise, strains were incubated at 37°C with shaking at 180 rpm. *S. aureus* strains were routinely grown in trypticase soy broth (Cat# HB4114, Hopebio) or on TSB agar plates. Luria Bertani (Cat# HB0128, Hopebio) broth and LB agar plates were always used for *E. coli* culture, and 50 μg/mL of kanamycin (Cat# A100408, Sangon Biotech) was used for plasmid maintenance. Nepetin (Cat# JOT -11737, Chengdu Pufei De Biotech Co., Ltd., 98% purity) was dissolved in DMSO at a concentration of 20 mg/mL. Other chemicals were purchased from Sinopharm Chemical Reagent Co. Ltd., China.

### Protein expression and purification

The BL21 (DE3) *E. coli* strain containing recombinant plasmid pET28a-clpP was the expression host. The cells were cultured in LB media containing 50 μg/mL kanamycin, and protein expression was induced with .5 mM Isopropyl-β-d-1-thiogalactopyranoside (IPTG) overnight at 20°C after OD_600_ reached .6. Afterward, the bacteria were resuspended in lysis buffer (40-mM Tris, pH = 8.5) and sonicated at low temperature for lysis. The lysate was cleared by centrifugation at 18,000 g for 1 h at 4°C and the supernatant was applied to His-Trap column (GE Healthcare). The obtained ClpP protein was desalted through an ultrafiltration concentration tube (Millipore), and the buffer was changed to ClpP buffer (100 mM HEPES and 100 mM NaCl, pH = 7.0) for use in subsequent experiments. The mutant proteins were expressed and purified following the same protocol as that for the wild-type protein.

### Screening of ClpP inhibitors

The screening of ClpP inhibitors was performed as previously described [21]. Suc-LY-AMC (Cat# S1153, Sigma–Aldrich) was the specific fluorescence substrate of ClpP, which was used to verify the hydrolytic activity of ClpP and screen the ClpP inhibitor [8,22]. Briefly, Chinese medicine monomer compounds and ClpP (final concentration 10 µM) were added to ClpP buffer and incubated for 1 h in a black 96-well plate. Then, 100 µM fluorescent substrate was added, and the fluorescence intensity (excitation: 360 nm, emission: 465 nm) was measured using an Infinite M200 instrument (TECAN) after a 1 h incubation at room temperature.

### Growth assay

To investigate the inhibitory effect of quercetin on the growth of *S. aureus*, overnight culture of USA300 was inoculated into 5 mL of fresh TSB medium supplemented with 100 µM of Nepetin or an equal volume of DMSO. The absorbance values at OD_600_ were measured every 1 h to generate growth curves.

### Cytotoxicity assay

The cytotoxicity of nepetin toward Vero cells (African green monkey kidney cell line) was assayed using MTT according to the manufacturer’s instructions. Briefly, Vero cells (8 × 10^3^ cells/well) were seeded into 96-well plates for culture. After 24 hours, they were treated with 100 μL of drug-containing medium for 24 hours. Afterward, the medium was replaced with 100 μL of MTT solution (final concentration .5 mg/mL) and incubated at 37°C for 4 h. The MTT solution was removed, MTT formazan was dissolved with 100 μL of DMSO, and the optical density (OD) values at 490 nm were measured.

### Quantitative real-time PCR

*S*. *aureus* USA300 was cultured in TSB containing 100 µM nepetin. A total of 5 × 10^8^ CFUs of bacteria were collected, and total RNA was extracted using TRIzol (Cat# B511311, Sangon Biotech). The cDNA templates were synthesized from total PrimeScript RT Reagent Kit (Cat# RR047Q, TaKaRa) and an ABI 7900HT Real-time PCR system was used for the qPCR analysis. Each test was performed three times independently. The primers used for qPCR are listed in Table S1. The DMSO-treated group was used as a control and 16S rRNA as an internal reference gene.

### Western blot analysis

The SDS–PAGE gel was transferred to a PVDF membrane and blocked with TBS supplemented with .1% (v/v) Tween-20 and 5% (w/v) skim milk powder. The rabbit anti-α-toxin polyclonal antibody (diluted 1:10,000) (Cat# S7531, Sigma–Aldrich) and the rabbit polyclonal anti-PVL LukS subunit (.5 μg/mL, Cat# ab190473, Abcam) were used to examine α-toxin and PVL levels, respectively. The HRP-labeled goat anti-rabbit IgG secondary antibody (diluted 1:2,000) (Cat# SE134, Solarbio) was incubated with the membrane. The gray values of bands on Western blots were calculated using ImageJ software.

### Hemolysis assay

*S*. *aureus* was cultured in TSB medium in the absence or presence of nepetin (4, 8, 16 and 32 μg/mL) until the OD_600_ reached 2.5. Cultures were centrifuged (5,500 g, 4°C 3 min) to collect the supernatant, which was filtered through a .22 µm filter. Afterward, 100 µL of supernatant were mixed with 875 µL of sterile PBS, and then 25 µL of de-fibrinated rabbit blood were added. The samples were incubated for 1 h at 37°C centrifuged at 5,500 g for 1 min at room temperature, and then the absorbance of the supernatant was measured at 543 nm. In addition, PBS without supernatant was used as a positive control group.

### Thermal shift assay (TSA)

TSA was essentially done as previously described [23]. SYPRO Orange is an environmentally sensitive dye that binds to the hydrophobic region of the protein and can therefore be used to detect the *T*_*m*_ value of the protein. ClpP (final concentration, 2 µM), SYPRO Orange (Cat# S5692, Sigma–Aldrich), nepetin and TSA buffer (10 mM HEPES and 150 mM NaCl, pH = 7.5) were all added to the opaque 96-well plate. The*Bio-rad iQ5* (Bio-Rad) heated the 96-well plate from 25°C to 90°C at a rate of 1°C/min.

### Cellular thermal shift assay (CETSA)

ClpP proteins were expressed using the same method, and the supernatant of the lysate was collected by centrifugation after ultrasonic fragmentation. Nepetin at 200 μM and an equal volume of DMSO were incubated with the supernatant at room temperature for 1 h, after which the insoluble precipitate was removed by centrifugation again (20,000 g, 20 min, 4°C). The resulting solution was aliquoted into PCR tubes and heated for 5 min at the indicated temperature in a Mastercycler® nexus gradient (Eppendorf) temperature cycler, followed by immediate immersion in an ice bath for 3 min. The samples were centrifuged to remove insoluble material and taken for western blot analysis. 6x-His Tag Antibody (Cat# MA1– 21315, Invitrogen) was used to detect ClpP protein, and the relative intensities of the target proteins were analyzed using ImageJ software.

### Localized surface plasmon resonance (LSPR)

Open SPR instruments (Nicoya, Canada) were used to analyze the interactions between nepetin and ClpP. The signal was first equilibrated using PBS (pH = 7.4) containing 1% DMSO, followed by activation of the chip by imidazole and NiCl_2_ to complete the functionalization of the chip surface. Next, the ClpP protein was immobilized on the chip, different concentrations of nepetin were injected after signal stabilization, and the binding and dissociation times of protein and ligand were both 240 s. Finally, the results were analyzed using TraceDrawer (Ridgeview Instruments ab, Sweden) and One To One.

### Urease activity

Urease agar base mediu (Cat# HB4095, Hopebio) containing the indicator phenol red was used to detect urease activity. Bacteria were inoculated into urease agar base medium and 100 μM nepetin was added. The color of the medium was observed after 1 day of incubation at 37°C. The production of pink color indicated a change in pH due to urea breakdown. Δ*clpP*-USA300 was used as a positive control.

### Molecular docking (MD)

Molecular docking was performed using Autodock vina 1.1.2 to investigate the binding mode of nepetin to ClpP [24]. The 3D structures of ClpP (PDB ID:3v5e) and quercetin (PubChem CID: 5317284) were obtained through the Protein Data Bank (www.rcsb.org) and PubChem database, respectively. The AutoDockTools 1.5.6 package was used to generate docking input files. Amber 14 and AmberTools 15 software performed molecular dynamics simulations to correct the molecular docking results [25]. Detailed parameters and procedures were described in a previous study [26].

### Pneumonia model experiment

The intranasal *S. aureus* infection model (pneumonia model) was developed as previously described [27]. Briefly, the 7-week-old female C57BL/6J mice were anesthetized with ether, and the mice were intranasally inoculated with the *S. aureus* suspension to infect the lung tissues. To evaluate survival, mice were infected with 2 × 10^8^ CFU/30 µL of *S. aureus* and treated with 100 mg/kg of nepetin administered subcutaneously 1 h and 12 h after infection. Mice were observed daily for survival and survival curves were generated. For histopathology and lung bacterial load analysis, 1 × 10^8^ CFU/30 µL *S. aureus* was used to infect mice, followed by subcutaneous injection of 100 mg/kg quercetin for treatment at 1 h and 12 h. After 24 h, mice were euthanized, lung tissues were excised under aseptic conditions, and the left lung tissue was homogenized and measured for CFUs. The right lung tissue was fixed with 10% formalin, and the tissue sections were stained with hematoxylin and eosin (H&E) for histopathological analysis.

### Statistical analysis

Each study was repeated three times independently. Statistical comparison between groups was analyzed using Student’s *t* test or one-way analysis of variance (ANOVA), and survival curve was analyzed using log-rank test. *P* < .05 were considered statistically significant.

## Results

### Screening of ClpP inhibitors

**Figure 1.**
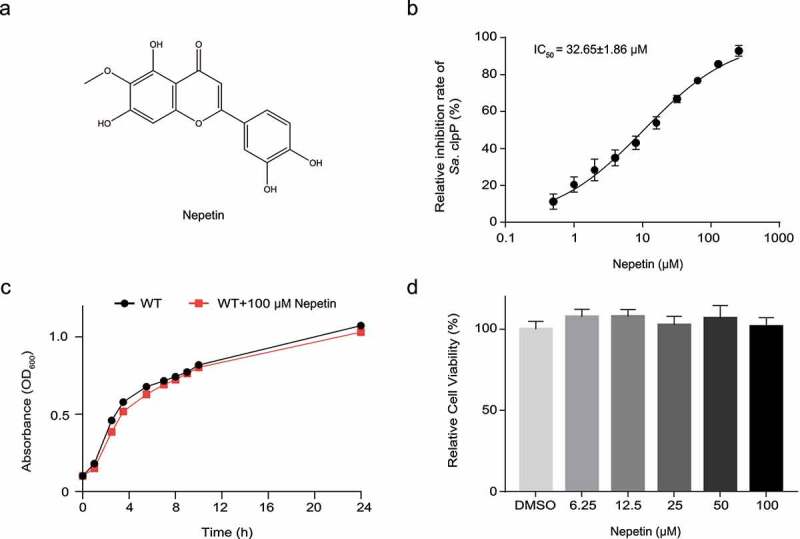
Nepetin inhibits the activity of ClpP and does not affect the growth of bacteria. (a) Chemical structural of nepetin. (b) Determination of IC_50_ values of nepetin on the fluorescent substrate Suc-LY-AMC.(c) The growth of USA300 is unaffected by 100 μM nepetin. Wild type USA300 was used as acontrol. 50 (d) Nepetin at 100 μM has no effect on the viability of Vero cells over 24 hours.

Natural herbal monomer compounds represent an important source of novel antibiotic agents [28]. We screened nepetin using Suc-LY-AMC, a specific fluorescent substrate for ClpP, in an existing chemical library of small molecules derived from natural products and determined an IC_50_ value of 32.65 ± 1.86 µM for nepetin inhibition of ClpP (Figure 1(a,b)). In order to investigate the manner in which nepetin inhibits ClpP activity, reversible experiments were performed. It was found that nepetin is a reversible inhibitor of ClpP and does not covalently bind to the active site of ClpP (Figure S1). The key to the antivirulence strategy is to inhibit the expression of virulence factors without affecting the growth of pathogenic bacteria. For this purpose, we determined the growth curve of nepetin against strain USA300 and showed that 100 µM of nepetin did not inhibit the growth of USA300 strain (Figure 1(c)). The results of subsequent MTT experiments showed that 100 µM nepetin had no effect on the proliferation of Vero cells (Figure 1(d)).

## Nepetin is a direct inhibitor of ClpP

**Figure 2.**
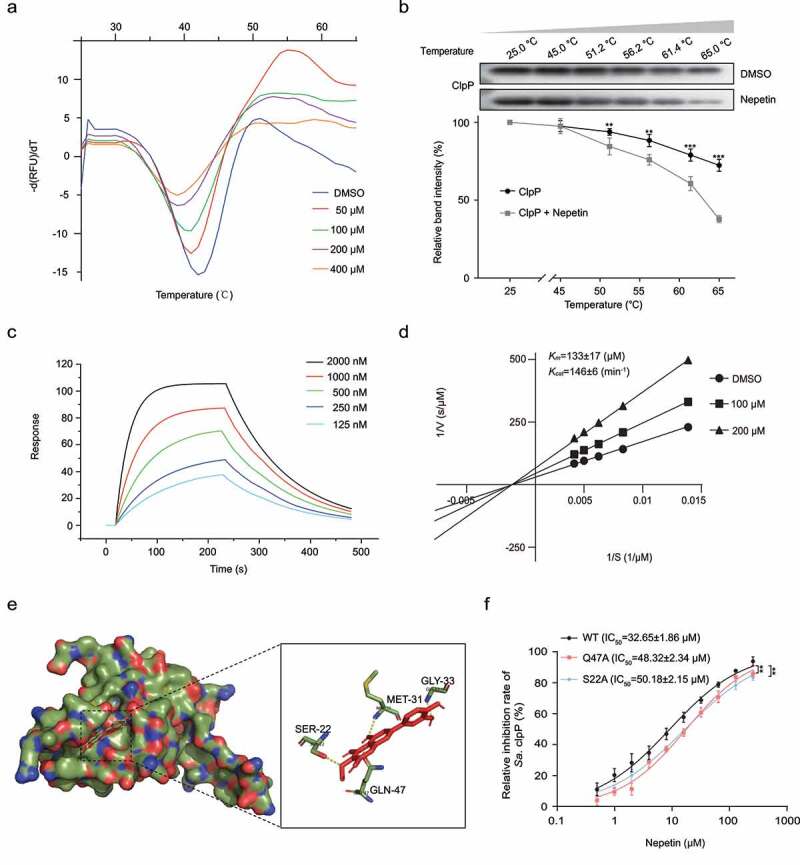
Nepetin binds to and inhibits ClpP activity. (a) the binding between nepetin and ClpP was examined by TSA. Nepetin caused a decrease in the thermal stability of ClpP. (b) western blot image and thermal shift assay curves showing that nepetin caused a decrease in the Tm of ClpP protein in the cell lysates of E. coli carrying pEt28a-clpP. Full western blot images are shown in Supplementary Fig. S3. (c) LSPR analysis demonstrates the kinetics of nepetin binding to ClpP. (d) Noncompetitive inhibition of nepetin on ClpP cleavage of Suc-LY-AMC. (e) Molecular docking predicted the binding pattern of nepetin and ClpP. (f) IC_50_ assay of nepetin on WT-ClpP and two mutants Q47A-ClpP and S22A-ClpP. the two mutant proteins showed some degree of resistance to nepetin inhibition. Significance is calculated based on two-tailed t-test: **P < .01 and ***P < .001.

TSA has been used to study the changes in the thermal stability of proteins after the binding ligand small molecules to proteins [23]. We analyzed the relationship between nepetin and the thermal stability of ClpP by performing TSA experiments to verify the direct interaction of nepetin and ClpP (Figure 2(a)). The thermal stability of ClpP became more unstable with increasing nepetin concentration, indicating that nepetin decreased the thermal stability of ClpP by interacting with the protein. The intracellular binding of nepetin to the ClpP protein was further observed using CETSA [29], and similar results were obtained with that of TSA. Nepetin was able to bind to ClpP and make ClpP thermally unstable (Figure 2(b)). We experimentally verified the binding behavior of nepetin and ClpP using an LSPR analysis to further investigate the mode of action of nepetin (Figure 2(c)). The association rate constant *K*_*a*_ and the dissociation rate constant are 1.88 × 10^4^ M^−1^s^−1^ and 3.70 × 10^−3^ s^−1^, respectively, within the hierarchy of ligand–protein interactions [30]. The equilibrium dissociation constant (*K*_*D*_ = 6.02 × 10 ^−7^M) indicated that ClpP and nepetin possesses a very strong binding capacity. Further kinetic studies showed that nepetin did not affect the Michaelis constant (*K*_*m*_ = 133 ± 17 µM) in the enzymatic reaction of ClpP and decreased the maximum velocity (*V*_*max*_), indicating that nepetin is a noncompetitive inhibitor of ClpP (Figure 2(d)). Altogether, these results show that nepetin is a direct inhibitor of ClpP.

## Molecular modeling and mutagenesis study

Molecular docking has become an important tool for studying the interaction between biomolecules and inhibitors, providing details of the binding of inhibitors to protein molecules at the molecular level [31]. We explored the structural basis of nepetin binding to ClpP by performing molecular docking to investigate the binding mechanism. The results are shown in Figure 2(e), residues Ser-22 have strong hydrogen bonding interactions with the ether linkage of nepetin. Met-31 and Gly-33 form strong hydrogen bonding interactions with the hydroxyl group of nepetin due to their close proximity to each other. Detailed analysis showed that residue Gln-47 formed a strong hydrogen bonding interaction with nepetin. These interactions help nepetin to dock to the binding site of ClpP.

Based on the results of molecular docking, mutant proteins Q47A-ClpP and S22A-ClpP were constructed and expressed. We determined the IC_50_ of nepetin against mutant proteins Q47A-ClpP and S22A-ClpP by fluorescent substrate Suc-LY-AMC, which were 48.32 ± 2.34 µM and 50.18 ± 2.15 µM, respectively, much higher than WT-ClpP (32.65 ± 1.86 µM). Compared with the IC_50_ of wild-type ClpP, the two mutant proteins showed some resistance to the inhibitory effect of nepetin (Figure 2(f)). Thus, amino acid residues Ser-22 and Gln-47 are involved in the binding process of nepetin to ClpP, which is consistent with the results of MD simulations.

### Nepetin represses the virulence of S. aureus in vitro

**Figure 3.**
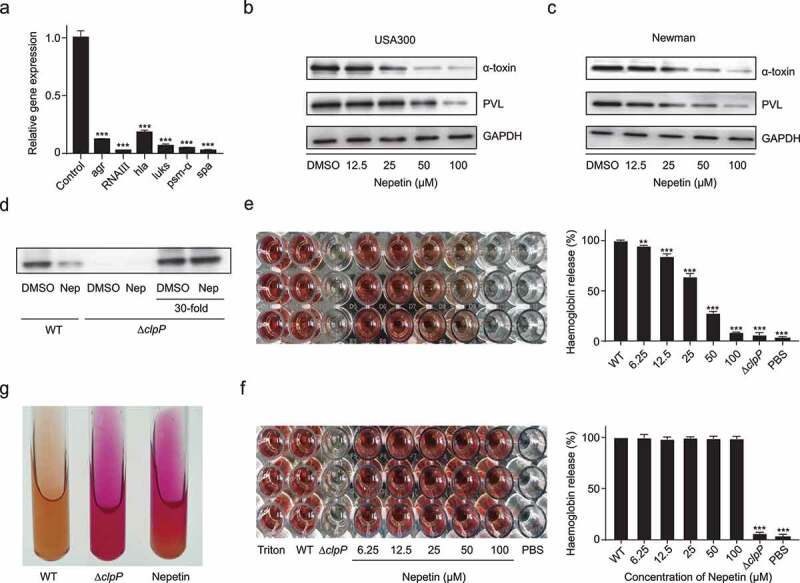
Expression of multiple virulence factors in S. aureus is inhibited by nepetin. (a) Expression levels of agr, RNAIII, hla, luks, psm-α and spa were determined by qPCR in the presence of 100 μM of nepetin. (b and c) Quantification of α-toxin and PVL expression levels in S. aureus USA300 and Newman under the effect of different concentrations of nepetin by western blot, and their corresponding gray value analysis. (d) The expression levels of nepetin on α-toxin in USA300 and Δclpp-USA300 were examined by Western blot. Nepetin lost its ability to inhibit α-toxin in Δclpp-USA300. (e and f) the effect of different concentrations of nepetin on the hemolytic capacity of S. aureus USA300 and USA300 supernatant. (g) Nepetin induces urease production, which causes the medium to show a pink color. Δclpp-USA300 was used as a positive control. Significance is calculated based on one-way ANOVA: **P < .01 and ***P < .001.

ClpP is closely related to the virulence of *S. aureus* [8]. Therefore, we detected the effect of 100 µM nepetin on the transcript levels of RNAIII, hla, luks, psm-α and spa using qPCR. As shown in Figure 3(a), levels of the *agr* and *RNAIII* transcripts, which are central to virulence regulation in *S. aureus*, were 10-fold and 50-fold decreased, respectively. The downregulation of important virulence factors, such as *hla*, *luks*, *psm-α* and *spa*, was evident, indicating that nepetin effectively inhibited the transcription of various virulence factors. Additionally, nepetin had no effect on the transcript levels of ftsz (a gene unrelated to virulence), which may suggest that the effect of nepetin on *S. aureus* is focused on virulence (Figure S2). Hemolysin (α-toxin) and leukocidin (PVL) mediate many different types of cell death and play an important role in the pathogenesis of *S. aureus*. We measured the expression of α-toxin and PVL in supernatant from MRSA strain USA300 and MSSA strain Newman treated with different concentrations of nepetin using Western blotting. Nepetin effectively inhibited the expression of α-toxin and PVL in a concentration-dependent manner (Figure 3(b,c)). The expression level of α-toxin was substantially down-regulated in *clpP* knockout *S. aureus*, but did not completely resolve, and the presence of α-toxin could still be detected after concentrating the supernatant. Analysis by western blotting revealed that nepetin had no effect on α-toxin production in Δ*clpP*-USA300, this result unequivocally indicated that nepetin inhibited the virulence of *S. aureus* through ClpP (Figure 3(d)). Since hemolysin exerts a significant hemolytic effect on rabbit erythrocytes [32], we used defibrinated rabbit blood to investigate the inhibitory effect of nepetin on the hemolysis of *S. aureus*. As shown in Figure 3(e), nepetin effectively inhibited the hemolytic capacity of *S. aureus*, which was consistent with the previous results of western blots. On the other hand, we incubated nepetin with the supernatant of the USA300 culture medium and found that nepetin did not inhibit the activity of α-toxin in the supernatant, indicating that the ability of nepetin to inhibit hemolysis was achieved by altering the expression of α-toxin rather than its activity (Figure 3(f)). ClpP in *S. aureus* is capable of inhibiting urease production, and deletion of ClpP would result in a surge in urease expression [33]. We examined the expression level of nepetin on the urease of USA300 by urease agar base. The results showed that nepetin could inhibit the expression of urease, resulting in a pink color of urease agar base, which was similar to the results of *clpP* knockout bacteria (Figure 3(g)).

## Nepetin can protect mice from lethal MRSA infection

**Figure 4.**
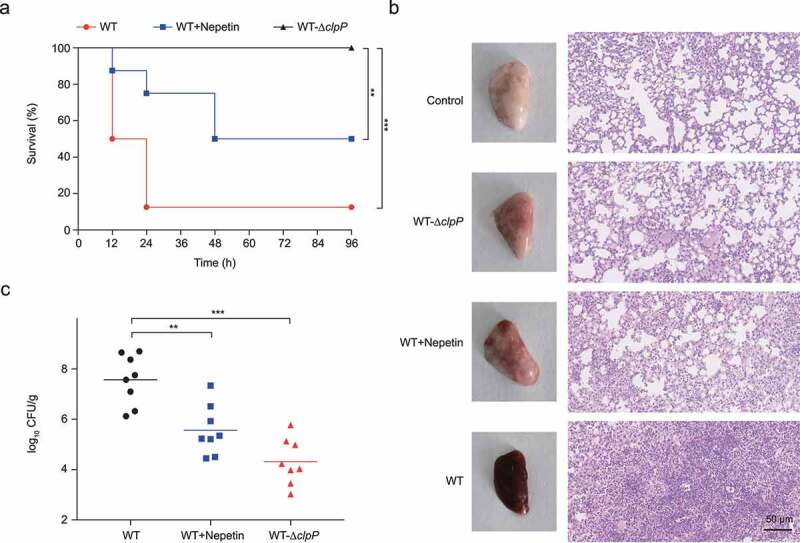
Nepetin protects mice from MRSA pneumonia. (a) Survival of mice treated with nepetin (100 mg/kg) at the indicated times after infection with USA300 (2e8 CFU/30 µl). Significance (p-value) in the panels except (a) is calculated using log-rank test: **P < .01 and ***P < .001. (b) Gross pathology and histopathology of *S. aureus* USA300 and USA300-Δclpp infected lung tissue from mice. Nepetin (100 mg/kg) treatment by subcutaneous injection. Scale bar, 50 μm. (c) the infectious bacterial load in the lung of mice with nepetin (100 mg/kg) treatment. in the graph, horizontal bars indicate the mean of bacterial load measurements, each dot represents a mouse. Significance is calculated based on one-way ANOVA: **P < .01 and ***P < .001.

*S*. *aureus* is a common cause of pneumonia and is associated with morbidity and mortality [34]. Our results have shown the ability of nepetin in reducing the virulence of MRSA *in vitro*. Therefore, we further evaluated the anti-infective effects of nepetin *in vivo* on a mouse pneumonia model. The survival of mice within 96 h was determined, and the results are shown in Figure 4(a). After intranasal inoculation with USA300, only 10% of mice eventually survived, while all mice inoculated with the Δ*clpP* knockout strain survived, which also implied that ClpP is important for the development of *S. aureus* pneumonia. After nepetin treatment, the survival rate of mice increased to 50%, suggesting that nepetin might exert a protective effect on mice with *S. aureus* pneumonia.

To further investigate the protective effect of nepetin in mice *in vivo*, we inoculated mice with 1 × 10^8^ CFUs of USA300 intranasally and treated them with the same dose of nepetin. At 24 h, all mice were euthanized, and lung tissue was collected for histopathologic analysis. As shown in Figure 4(b), the lung tissues of normal mice were light pink and elastic, with no inflammatory cell infiltration in the alveolar cavity, while the lung tissues of *S. aureus*-infected mice were dark red, diffuse hyperemia and edema. The results of H&E staining showed deformation of the alveolar wall after MRSA infection and massive infiltration of inflammatory cells into the alveoli. After nepetin treatment, the lung tissues of the mice showed a significant improvement in congestion, the number of inflammatory cells in the alveolar lumina was reduced, and the amount of intraalveolar edema and hemorrhage was decreased, indicating that inflammation in the mouse lungs was improved. Results of bacterial load measurement in the lungs of challenged mice revealed a decrease from 7.57 log_10_ CFU/organ to 5.56 log_10_ CFU/organ in the nepetin-treated group of mice, consistent with H&E staining results (Figure 4(c)). Overall, these results suggest that nepetin renders MRSA less pathogenic in this mouse infection model and protected mice from lethal infection with MRSA.

## Discussion

*S*. *aureus* has developed multiple mechanisms of resistance to almost all known antibiotics, and there is a great need for the identification of novel anti-infective agents to attack new targets to overcome drug resistance. Inhibition of virulence factor expression is considered a therapeutic strategy for *S. aureus* infection that targets the virulence rather than the viability of the bacteria and therefore does not exert strong selection pressure on the bacteria and reduces the development of drug resistance. In addition, the antivirulence strategy is more advantageous because it does not harm the normal microflora in the host body like antibiotics [35]. Natural products are not only vital medicinal resources but also a source for the detection of novel antitoxicant drugs and even antibiotics, and a variety of natural products have been shown to exert good antitoxicant effects [13].

ClpP may regulate the transcription of multiple virulence factors in *S. aureus* by regulating *agr* and *mgrA* [10,36]. The Agr system has long been recognized as a key regulator of virulence expression in *S. aureus* that is capable of initiating the expression of downstream virulence factors via RNAIII [37]. Here, we show that nepetin significantly reduces the level of the *agr* transcript, which may be achieved by inhibiting ClpP activity. Alpha-toxin is the most studied virulence factor of *S. aureus*, which binds to target cell membranes and causes cell damage [38]. Meanwhile, the key role of PVL and α-toxin in the pathogenesis of *S. aureus* has been confirmed in laboratory animals [39]. Our results suggest that nepetin effectively suppresses the expression of PVL and α-toxin and thus reduce the virulence of *S. aureus*. ClpP protease is important for the development of *S. aureus* pneumonia [40]; therefore, we constructed a pneumonia model of *S. aureus*-infected mice and treated them with a subcutaneous injection of nepetin. Nepetin effectively ameliorated congestion and inflammation in the mouse lungs and significantly reduced the bacterial load in the mouse lungs. These experimental results suggest that nepetin protects mice from lethal pneumonia infection with MRSA by inhibiting ClpP activity and subsequently downregulating the expression of PVL and α-toxin, which ultimately abrogates the pathogenicity of *S. aureus* in mice.

The direct modulation of target protein activity by small molecule compounds lies in the ability to bind to the protein [30]. We performed TSA and CETSA and showed that nepetin binds to ClpP and reduce its thermal stability. The LSPR technique is considered one of the most important tools for analyzing biomolecular interactions and is able to determine several kinetic and affinity parameters. We determined the equilibrium dissociation constant of 602 nM for the interaction between nepetin and ClpP by LSPR, indicating binding between nepetin and ClpP. Most importantly, nepetin had no effect on α-toxin expression in Δ*clpP*-USA300, suggesting that nepetin attenuates the virulence of *S. aureus* in a ClpP-dependent manner. In addition, although nepetin has pan-assay interference structures (PAINS) moiety catechol, our above experiments have shown that nepetin could strongly bind to ClpP and inhibit ClpP activity. Meanwhile, nepetin effectively attenuated the virulence of *S. aureus* by inhibiting ClpP and effectively fought against MRSA infection. Therefore, we believe that nepetin is a compound with powerful anti-*S. aureus* virulence.

The virulence of *S. aureus* is regulated by multiple regulators, and inhibition of the expression of one virulence factor is not effective in suppressing the pathogenicity of *S. aureus*. Therefore, simultaneous inhibition of multiple virulence factors would be a promising strategy. Since ClpP plays a central regulatory role on the virulence of *S. aureus*, inhibition of ClpP may be effective in limiting the pathogenicity of *S. aureus* [8]. Nepetin may also inhibit other virulence regulators, but nepetin reduces *S. aureus* virulence by inhibiting ClpP activity, providing new insight into the ability of nepetin to reduce the virulence of *S. aureus*. In conclusion, we found that nepetin protected mice from lethal pneumonia infection with MRSA by inhibiting ClpP, and it may be further developed as a potential antimicrobial agent.

## Ethical statement

Animal experiments were conducted in strict compliance with the ARRIVE guidelines and the guidelines of the Animal Ethics Committee of Changchun University of Chinese Medicine.

## Supplementary Material

Supplemental Material

## Data Availability

All data generated during this study are available on request from the corresponding authors.
