# Supplementary material for: Nepetin reduces virulence factors expression by targeting ClpP against MRSA-induced pneumonia infection
Source: Virulence. 2022 Apr 1;13(1):578–88. doi: 10.1080/21505594.2022.2051313 (PMC8986306; doi:10.1080/21505594.2022.2051313)
Supplement: Supplemental Material [file KVIR_A_2051313_SM1219.doc]

**Supplementary Information**

**Nepetin reduces virulence expression by targeting ClpP against MRSA-induced**

**pneumonia infection**

Shisong Jing^1^, Xinran Ren^1,2^, Li Wang^1^, Xiangri Kong^3^, Xingye Wang^1,5^, Xiren Chang^1,3^, Xuerui Guo^2^, Yan Shi^2^, Jiyu Guan^6^, Tiedong Wang^4^, Bingmei Wang ^1^*, Wu Song^1^*, and Yicheng Zhao^1^*

1. Clinical Medical College, Changchun University of Chinese Medicine, Changchun 130117, China.

2. School of Pharmaceutical Science, Jilin University, Changchun 130021, China.

3. Affiliated Hospital to Changchun University of Chinese Medicine, Changchun University of Chinese Medicine, Changchun 130021, China.

4. College of Animal Science, Jilin University, Changchun 130062, China.

5. College of integrated Chinese and Western medicine, College of rehabilitation, Changchun University of Chinese Medicine, Changchun 130117, China.

6. Key Laboratory of Zoonosis, Ministry of Education, College of Veterinary Medicine, Jilin University, Changchun 130062, China.

* Correspondence: Y.C. yichengzhao@live.cn; W.S. five841110@126.com; B.M. bingmeiwang1970@163.com.

**Table S1: Primers used in this study**

**Figure S1. Reversible inhibition**

**Figure S2. Detection of transcript levels of ftsz by qPCR**

**Figure S3. The full western blot images of CETSA**

**Table S1: Primers used in** this study

| Primer name | | Sequences (5´-3´) |
| --- | --- | --- |
| rt-*RNAIII*-f  rt-*RNAIII*-r  rt-*hla*-f  rt-*hla*-r  rt-*spa*-f  rt-*spa*-r  rt-*agr*-f  rt-*agr*-r  rt-*lukS*-f  rt-*lukS*-r  rt-*psm*-f  rt-*psm*-r  rt-*ftsZ*-f  rt-*ftsZ*-r  rt-*16S rRNA*-f  rt-*16S rRNA* -r | GCACTGAGTCCAAGGAAACTAAC  AAGCCATCCCAACTTAATAACC  AAAAAACTGCTAGTTATTAGAACGAAAGG  GGCCAGGCTAAACCACTTTTG  CAGCAAACCATGCAGATGCTA  GCTAATGATAATCCACCAAATACAGTTG  GCCCATTCCTGTGCGACTTA  GGGCAAATGGCTCTTTGATG  GAGGTGGCCTTTCCAATACAAT  CCTCCTGTTGATGGACCACTATTA  TATCAAAAGCTTAATCGAACAATTC  CCCCTTCAAATAAGATGTTCATATC  TGCAATCCAAGGTGCAGACA  CACCAACAGTTAATGCGCCC  GCTGCCCTTTGTATTGTC  AGATGTTGGGTTAAGTCCC | |

rt refers to qPCR primers.

**Figure S1. Reversible Inhibition**

The 10-fold IC_50_ concentration of nepetin was incubated with 100 µL of ClpP (100 µM) for 1 h at room temperature and then diluted by adding 9.9 mL of reaction buffer. After that, 90 µL of the mixed solution was added to a black opaque 96-well plate with the substrate peptide Suc-LY-AMC to a final concentration of 10 µM. The fluorescence intensity (excitation: 360 nm, emission: 465 nm) was measured using an Infinite M200 instrument (TECAN).

**
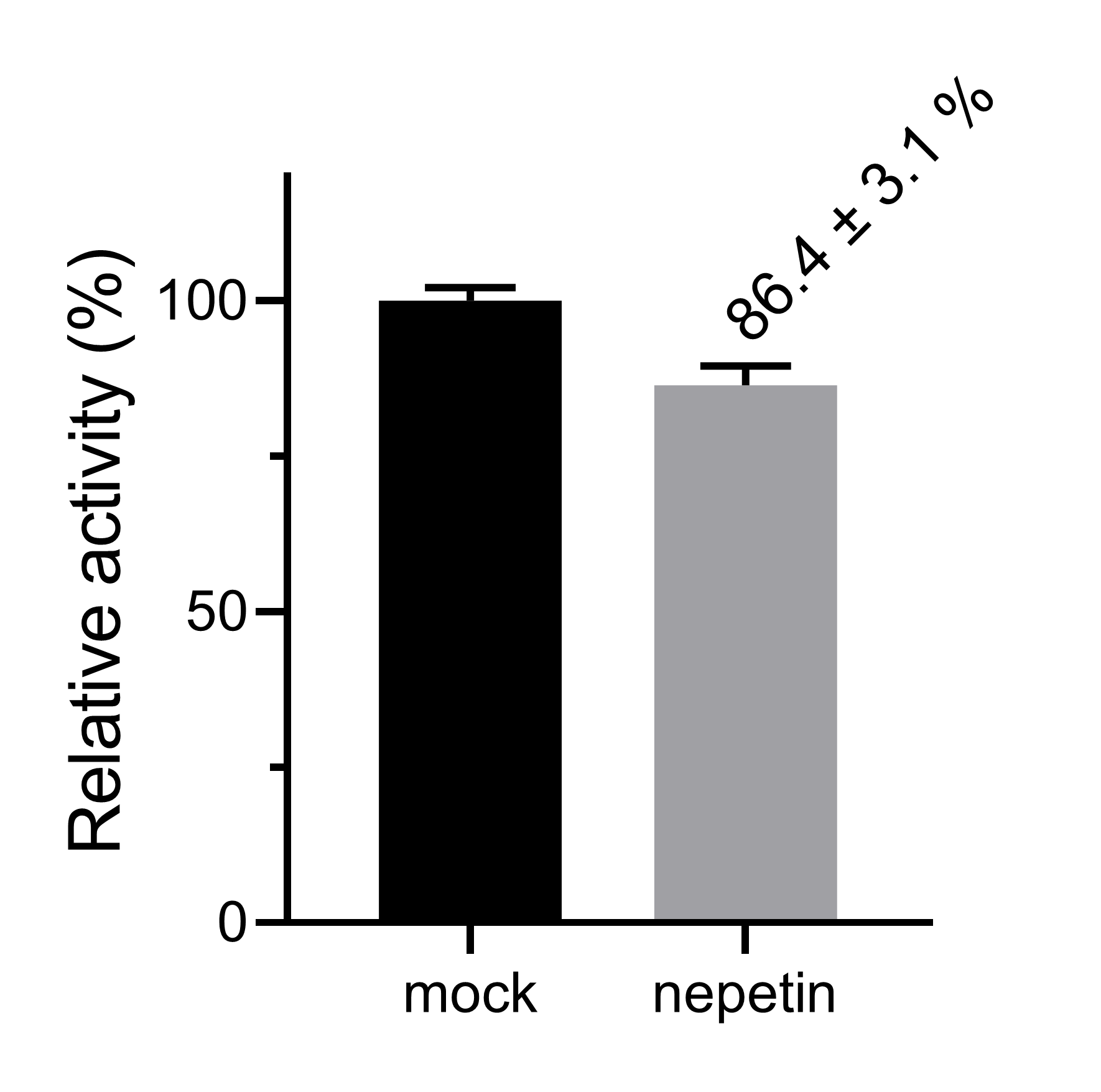
**

**Figure S1.** ClpP was incubated with buffer alone or with compound nepetin at 10 × IC_50_ concentration and diluted, then ClpP activity was measured by Suc-LY-AMC cleavage. Control (mock) sample was assigned 100% activity. Eighty-six percent (±3.1) activity were recovered from ClpP treated with nepetin inhibitor.

**Figure S2. Detection of transcript levels of ftsZ by qPCR**

**
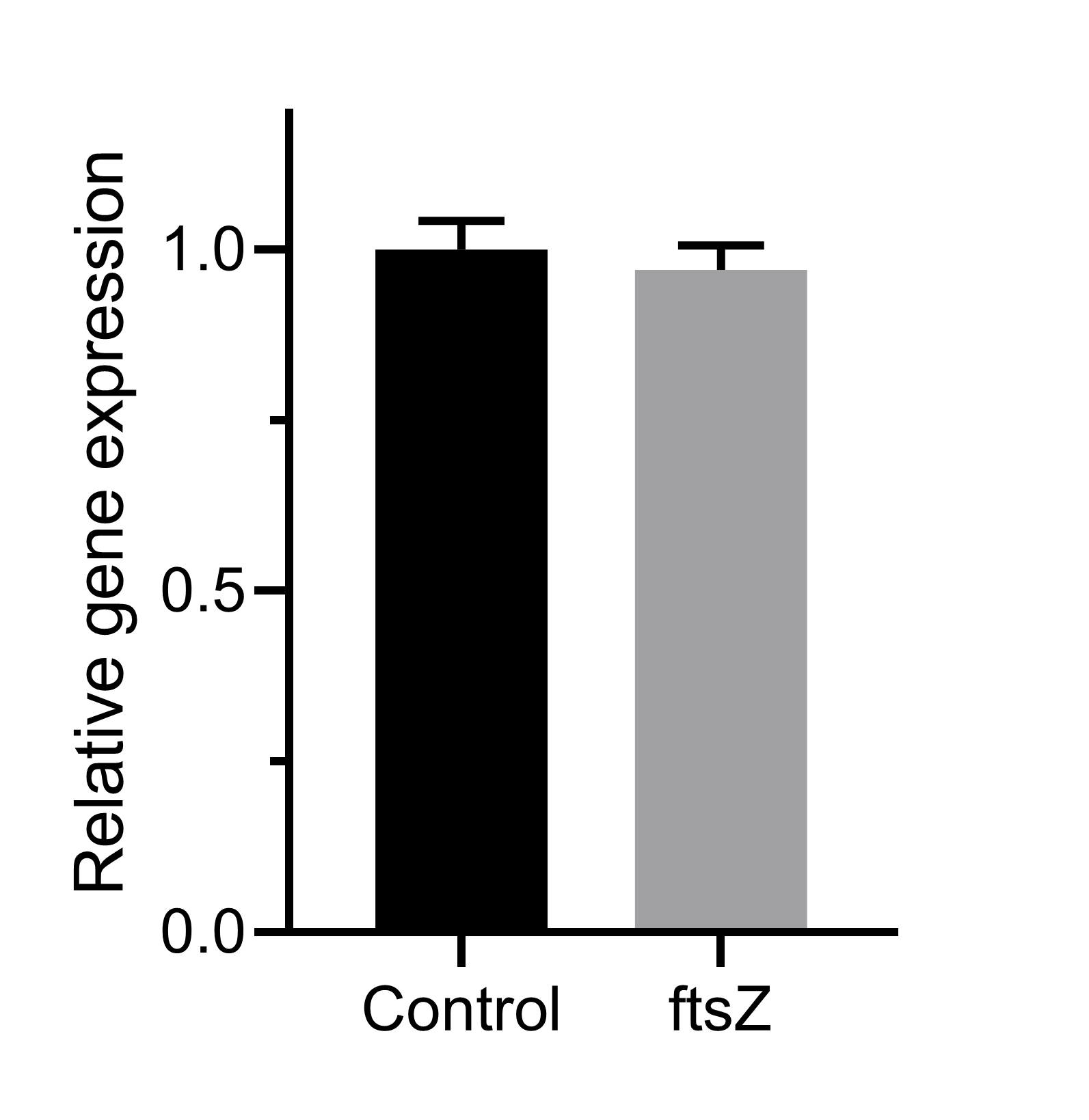
**

**Figure S2.** The transcript levels of ftsZ in nepetin-treated USA300 strains were determined by qPCR. The results showed that ftsZ was not affected by nepetin, suggesting that nepetin may primarily act on the virulence target of *S. aureus*. The DMSO-treated group was used as a control, and 16S rRNA was used as an internal reference gene.

**Figure S3. The complete western blot images of CETSA**

**
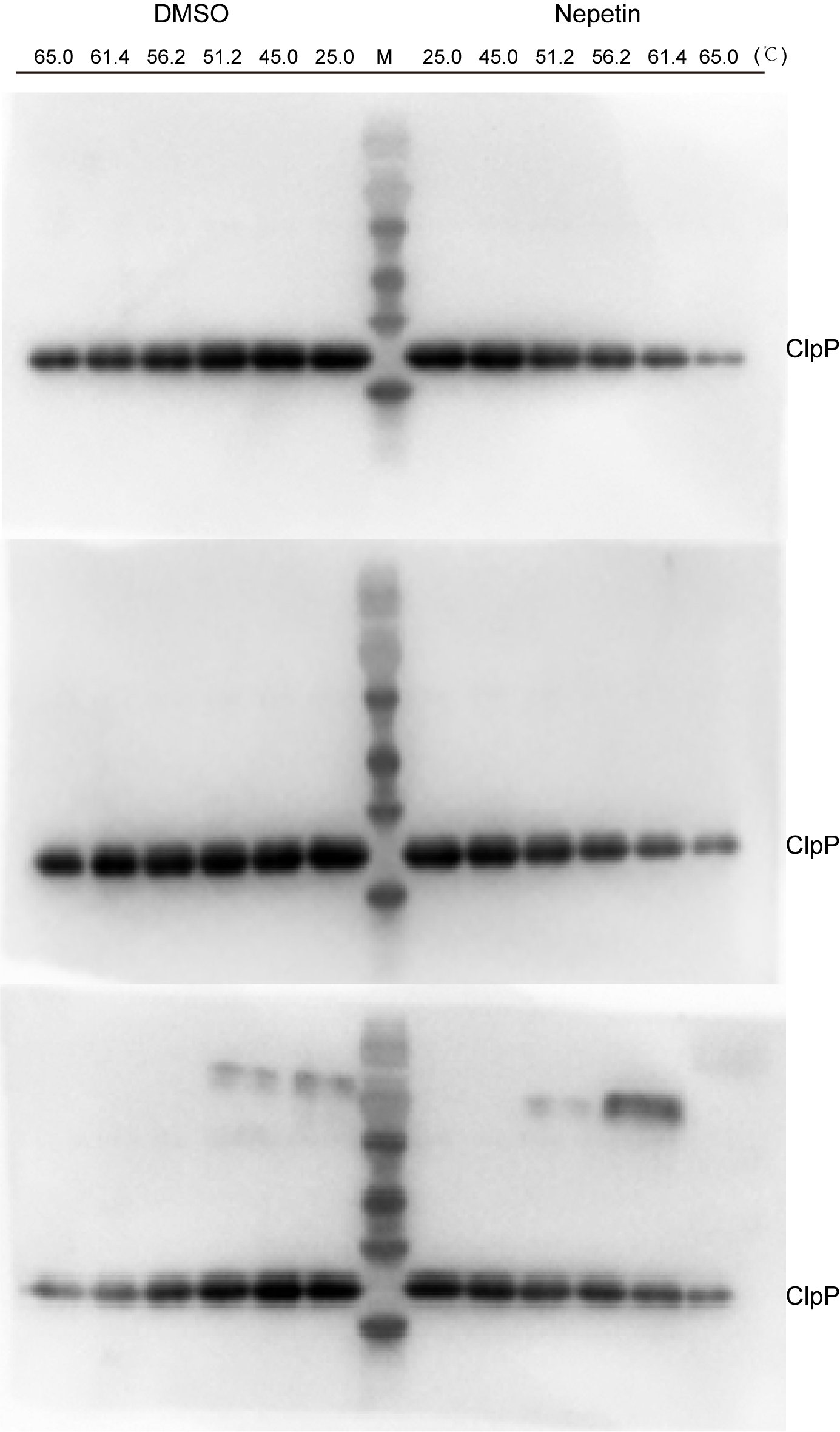
**

**Figure S3.** The complete western blot images of CETSA. Uncut western blot images illustrated that nepetin (200 μM) decreases the *T_m_* value of ClpP protein in Rosetta-pET28a–clpP cells. Experiments were performed on three independent occasions, as indicated.
